# SMART Multi-Criteria Decision Analysis (MCDA)—One of the Keys to Future Pandemic Strategies

**DOI:** 10.3390/jcm14061943

**Published:** 2025-03-13

**Authors:** Gianina-Valentina Băcescu Ene, Mirela-Anca Stoia, Cristian Cojocaru, Doina Adina Todea

**Affiliations:** 1 Department of Pneumology, “Iuliu Hațieganu” University of Medicine and Pharmacy, 400332 Cluj-Napoca, Romania; bacescu.ene.giani.valentina@elearn.umfcluj.ro (G.-V.B.E.); dtodea@umfcluj.ro (D.A.T.); 2 Department of Internal Medicine, “Iuliu Hațieganu” University of Medicine and Pharmacy, 400332 Cluj-Napoca, Romania; mirelastoia@yahoo.com; 3 Department of Cardiology, Emergency County Clinical Hospital, 400006 Cluj-Napoca, Romania; 4 Medical III Department, Faculty of Medicine, “Grigore T. Popa” University of Medicine and Pharmacy, 700115 Iasi, Romania

**Keywords:** COVID-19, Multi-Criteria Decision Analysis (MCDA), Multi-Criteria Decision Making (MCDM), performance matrix, The Simple Multi-Attribute Rating Technique (SMART), criteria/key attribute (risks and opportunities), alternatives, patient-centered decision making, healthcare professionals’ preferences, scenario simulations

## Abstract

**Background/Objectives:** The COVID-19 pandemic underscored the need for adaptive public health strategies and effective decision-making tools to optimize clinical responses and policy measures based on regional contexts. This study aims to identify key criteria for developing a patient-centered strategy to enhance the resilience of Romania’s healthcare system during the pandemic. **Methods:** This research introduces a Multi-Criteria Decision Analysis (MCDA) model using the Simple Multiple Attribute Assessment Technique (SMART) to integrate quantitative and qualitative data, providing decision-makers with a structured tool for improving healthcare resilience. A survey of 412 Romanian healthcare professionals identified critical risks and opportunities. The study followed a two-phase approach: first, analyzing expert perceptions to determine key challenges; second, applying a mixed-methods evaluation to prioritize resilience-building strategies. **Results**: Four main challenges emerged: (1) healthcare workforce shortages causing excessive workload and stress, (2) poor communication and systemic inefficiencies limiting patient access, (3) weak crisis management due to delayed control measures, and (4) regulatory gaps leading to fragmented responses. Proposed solutions included workforce training, improved communication, telemedicine integration, increased financial support, and a unified legal framework. The SMART method facilitated the structured prioritization of these measures, with long-term system sustainability emerging as the most effective strategy for preventing future crises. **Conclusions:** This study demonstrates the value of integrating MCDA into healthcare decision-making, offering a scalable model for policymakers to enhance crisis response and resource allocation. By incorporating expert insights and patient needs, the proposed framework strengthens healthcare system preparedness, contributing to informed, patient-centered decision-making and long-term resilience. Ultimately, our findings not only contribute to the existing literature but may also open new directions to facilitate informed, patient-centered decision making, thereby strengthening the resilience of healthcare systems in crisis situations.

## 1. Introduction

The unique challenges posed by the COVID-19 pandemic, along with the limited data on SARS-CoV-2 during the state of emergency and alert, created a pressing need to prioritize and rapidly implement measures to mitigate the outbreak’s effects. This context inspired us to employ the MCDA method to identify the most effective strategies for enhancing the resilience of the Romanian healthcare system, leveraging the perceptions, attitudes, knowledge, and expertise of healthcare professionals during the pandemic. Still, these tools should be tailored to the specific region or country of application, in order to adequately address potential risk factors, threats, or opportunities while considering local priorities, resources, preferences, and needs.

In the healthcare sector, MCDM has gained prominence due to its adaptability and flexibility, particularly in addressing complex decision-making scenarios. It is frequently employed for tasks such as cost-effectiveness analyses and the evaluation of new technologies or interventions, especially in contexts where limited data are available—such as rare diseases or oncology [1,2]—helping to benchmark health interventions based on criteria that extend beyond clinical effectiveness, including their impact on budgets and ethical considerations (Campolina et al., 2021) [3]

MCDA is a natural fit for patient-centered care as it can incorporate patients’ values and preferences into decision-making, comparing multiple competing options (e.g., healthcare interventions) based on their performance across multiple, often conflicting criteria [4]. The MCDA process generally involves the following: problem structuring (i.e., participant selection, alternatives, and criteria); MCDA modeling (i.e., weighting, scoring, and aggregation); and decision making (i.e., interpreting the results and making decisions) [5]. Nonetheless, implementing MCDA in clinical practice poses several challenges. Salajan et al. [6] have identified three key recommendations for improving decision-making in the context of infectious disease outbreaks: (1) Developing decision-making skills, (2) fostering collaboration among key actors, and (3) ensuring transparency. A key issue is the selection of relevant criteria and the assignment of weights, which may differ depending on the stakeholder group, such as clinicians, patients, or investors. In addition, the interpretation and communication of results can be difficult, especially in cases where the trade-offs are complicated or the results are not obvious [7].

Gongora Salazar P. et al. (2023) [8] also observed that, although the application of MCDA continues to grow in different decision-making contexts in healthcare, the lack of quality and transparency is an obstacle to the widespread adoption of MCDA in healthcare. In the healthcare literature to date, we have not found any applied examples that could serve to evaluate the strategies that should be adopted to deal with future threats or situations, such as the COVID-19 pandemic, through adopting MCDA and linear additive models (Multi-attribute Utility Theory, weighted sum) [9]. However, strategic documents such as the National Health Strategy 2023-2030, developed by the Ministry of Health [10], emphasize the need for evidence-based and transparently prioritized health policies. Despite this, our review of the national literature did not find any published studies that explicitly apply MCDA to the Romanian health sector during the pandemic. Furthermore, while the study “Successes and Failures of the Initial Response to the COVID-19 Pandemic in Romania” by Ștefan Dascălu [11] provides a qualitative and descriptive analysis of Romania’s initial response—examining factors such as health infrastructure, public communication strategies, quarantine measures and population response—it does not apply a formal multi-criteria decision-making method. Furthermore, our broader literature review did not identify studies that integrate MCDA with the SMART method to determine the best alternative in contexts requiring urgent prioritization and emergency interventions. This gap highlights the need for further research to explore how these methodologies can improve systematic and effective decision-making in crisis situations.

Considering all these points, our research aims to bring attention to an empirical example that, through adopting MCDA and the SMART [12,13], involves evaluating and comparing the essential criteria obtained from a survey applied to 412 Romanian healthcare professionals in choosing the best strategy or alternative option to increase the resilience of the national healthcare system, focused on the needs of patients and healthcare professionals.

Consequently, the entire focus of the paper is centered on this question:

“*Could MCDA, using a linear mathematical approach that incorporates quantitative and qualitative data focused on patient needs and healthcare expert preferences, serve as a model to support policymakers in improving the resilience of healthcare systems?*”

In accordance with the concept of this study, the main objectives were: (1) identifying the essential criteria (risks and opportunities) and alternative solutions for optimizing and reforming the health system, and (2) identifying the most likely strategy/best alternative option for increasing the resilience of the national health system through adopting MCDA and using the SMART.

Consequently, the specific objectives highlighted in this study included identifying analytical criteria and alternative solutions for optimizing and reforming the healthcare system, as follows:

(a) Identifying factors that could influence the practice of healthcare professionals, as well the possible solutions/opportunities that should be implemented in the future to deal with similar situations.
(b) Identifying alternative solutions for optimizing patient accessibility to healthcare services, as well as the risks encountered by patients in accessing healthcare services during a state of emergency and alert.
(c) Identifying barriers/limits and the possible solutions (risks and opportunities) offered by healthcare professionals for a resilient healthcare system.

## 2. Materials and Methods 

This prospective study was conducted in accordance with the guidelines outlined in the STROBE statement [14,15].

This manuscript explores the optimization of public health strategies in the Romanian healthcare system during the COVID-19 pandemic using Multi-Criteria Decision Analysis (MCDA) and the Simple Multiple Attribute Assessment Technique (SMART). This methodology was used to evaluate the available options for increasing the resilience of the national healthcare system.

After a review of the specialized literature and the Web of Sciences core collection, we identified only 102 studies that have used multi-criteria analysis (MCDA) as a tool for evaluating health systems. Applying the filters and keywords MCDA healthcare system/MCDA healthcare system-COVID-19 and/or MCDA using the SMART, we identified only four studies that used MCDA and/or the SMART. Most of these studies focused on pharmacoeconomics, health policies, evaluation of health services, or management of the COVID-19 outbreak, such as the remarkable study titled “Composite Monte Carlo Decision Making under High Uncertainty of Novel Coronavirus Epidemic Using Hybridized Deep Learning and Fuzzy Rule Induction” [16], in which, the authors applied a composite Monte Carlo and Neuro simulation method, induction with fuzzy rules, to extrapolate available data and generate possible future scenarios for the epidemic. Of these studies, only two studies came close to our approach; namely, those of Abdullah et al. [17] and Ahmad et al. [18], in which Abdullah et al. used the SMART to show the usefulness of the method for price evaluation and policy formulation and strategic planning to improve the procurement of generic drugs, while Ahmad et al. used the BMW technique (i.e., a best vs. worst method) to prioritize criteria that had been chosen from literature for ten pre-defined COVID-19 outbreak mitigation and appropriate resource and medical services allocation strategies through comparing the alternatives in a pairwise manner (worst vs. best).

What differentiates this study from other research is the originality of the approach, which consists of applying a practical example that incorporated MCDA and the additive linear model (Multi-attribute Utility Theory, weighted sum) [9] (p. 2) and which, based on the criteria identified following a survey applied to 412 Romanian healthcare professionals, aimed to identify the strategy most likely to increase the resilience of the national healthcare system. Accordingly, the research was conducted in two stages. The first stage focused on an analysis of means, scales, rankings, and trends to identify criteria based on the perceptions of healthcare experts. The second stage involved the evaluation of the identified criteria using MCDA with the linear additive model [9] (p. 2), in order to determine the most effective strategy for increasing the resilience of the Romanian healthcare system.

### 2.1. Data Collection

The survey was distributed online (docs.google.com) from the 27 January 2022 to the 28 February 2022 via a link through social networks (WhatsApp/Facebook/Messenger healthcare professionals’ groups) and email.

### 2.2. Ethics Considerations

The research was conducted in accordance with the Declaration of Helsinki and approved by the Ethics Committee of the Iuliu Hațieganu of Medicine and Pharmacy Cluj-Napoca Romania (according to AVZ 6/26 January 2022). Given the nature of the research, based on the knowledge, attitude, and practices towards COVID-19 of medical staff, the decision to participate or not in this survey did not interfere in any way with the rights of participants as healthcare professionals and the participants gave their consent by completing the questionnaire. The confidentiality and anonymity of the participants were maintained throughout the study, and their identification data were not collected. They were informed that their participation was voluntary, and that they could withdraw from the study at any time.

### 2.3. Study Design

The manuscript attempts to provide a comprehensive assessment of risks and opportunities, as well as possible interventions or solutions, based on the needs of patients and healthcare professionals.

The research project is a quantitative observational study in which data were collected longitudinally through a digital questionnaire “Effects felt by healthcare professionals in Romania during the COVID-19 pandemic (both emergency and alert)” (Supplementary Materials File S1).

The resulting data were analyzed in two stages of analysis. The first stage of analysis (optimization of the data resulting from the survey) was divided into three sections. The first section collected the demographic characteristics of the participants and their level of knowledge and expertise in the field, which helped us to compare the responses. The second section was divided into two subsections: (1) Perceptions regarding knowledge, attitudes, and practices towards COVID-19; and (2) perceptions, practices, and preferences relating to increasing patient accessibility to healthcare services. The third section (qualitative section and feedback section) aimed to identify the limits and alternative solutions for increasing the resilience of the national healthcare system.

The second stage of analysis (optimization of data obtained in the first stage) involved integrating the identified criteria into a multi-criteria analysis and using the SMART technique to identify the optimal alternative or the most likely strategy to increase the resilience of the national healthcare system.

### 2.4. Population and Sample Size

A convenience sampling method was used to reach eligible participants among Romanian healthcare professionals. When calculating the sample size, we considered the target population (doctors, dentists, nurses, and pharmacists from the civil and private sectors in Romania, including the National Public Order and the National Security System). Eligible study participants received an invitation via email with information about the project and a web link to the survey, or via social media on the WhatsApp/Messenger group profiles of healthcare professionals. Participants were informed that the research approach fully complied with the legal provisions regarding the confidentiality of personal data GDPR and, by participating in the questionnaire, they would give their consent to participate, agreeing that the collected data would be subsequently used as statistical data. We considered all participants over the age of 21 who agreed to participate in the online questionnaire as eligible. Individuals who were unable to complete the questionnaire and were ineligible were excluded.

As no comparable studies from Romania or other countries that could be used to calculate the representative sample size were available, we used data provided by the National Institute of Population Statistics in 2021, which mentioned the activity in the Romanian healthcare system of 366,821 healthcare professionals [19]. For this reason, we applied the theories of Cochran (1963), Taro Yamane (1967), and P. Mureșan (1989) for large populations where the incidence of the phenomenon is unknown and the maximum value of the dispersion is 0.25 [20,21,22]. According to these theories, the proposed sample size for large populations (N ≥ 100,000 inhabitants) should be 384, considering a maximum dispersion value of 0.25, an error of 5%, and a confidence interval of 95%. In other words, the sample size providing a guarantee of 95% of the results with the maximum allowed error of 5% is 384.

The notoriety of the topic—considering the fake news, spam, and misinformation that included the COVID-19 pandemic as a topic—made it difficult to obtain a larger number of respondents. However, the representativeness of the sample was ensured and the total number of survey participants considered was 412, which remained above the proposed sample size with a response rate of 93.20% (384/412).

### 2.5. Statistical Analysis and Measurement Parameters

The data obtained from the survey was entered and validated using Excel from Microsoft Office Plus 2021. In the analysis, we relied exclusively on descriptive statistics—such as ranges, trends, means, medians, standard deviations and percentage indices—to compare questions with variable indices and did not perform correlation or regression analyses as we did not aim to test hypotheses, but only to identify essential criteria for increasing the resilience of the national health system. Categorical data are presented as ranking indices, rating scales, trends, percentages, and evolution indices. For continuous quantitative data, we use the mean rank and standard deviation (SD). Following the research design, for the questions that collected socio-demographic characteristics and qualitative feedback, we assigned different weights depending on the level of education, expertise, and involvement in pandemic control activities to achieve the distinction of the respondents’ answers. Therefore, differentiated weight coefficients (w1) were assigned accordingly:

(1) For the level of education, the value of the weight coefficients was distributed from 1 to 3.5, where 1 represented the minimum and 3.5 was the maximum weight, as follows:
  ▪ Health assistants: 1;
  ▪ Medical assistants: 1.5;
  ▪ Resident doctors: 2;
  ▪ Specialist doctors/pharmacy supervisors/pharmacists: 2.5;
  ▪ Primary doctors and primary pharmacists: 3;
  ▪ Ph.D. in medicine/pharmacy or academic field: 3.5.
(2) For the expertise in the field of the respondents (years of experience in the specialty), the value of the weight coefficient was distributed from 1 to 3, where 1 represented the minimum and 3 the maximum, as follows:
  ▪ Between 0 and 4 years: 1;
  ▪ Between 5 and 9 years: 1.5;
  ▪ Between 10 and 14 years: 2;
  ▪ Between 15 and 19 years: 2.5;
  ▪ Over 20 years: 3.
(3) For the experience acquired during the COVID-19 pandemic (number of missions, involvement or not in activities to prevent/mitigate the spread of infections), we assigned differential weight coefficients, where 1 represented the minimum and 3 the maximum, as follows:
  ▪ Those not involved/department/education: 1;
  ▪ Those in public health directorates/ministry/pharmacy/non-governmental organizations (NGOs): 1.5;
  ▪ Those in triage centers, vaccination centers/triage missions at border-crossing points: 2;
  ▪ Those in civil hospitals: 2.5;
  ▪ Those in COVID support hospitals/emergency care unit/ambulance: 3.

For identification of the essential criteria for enhancing the resilience of the national healthcare system, we used several Likert-type point scales [23] to assess participants’ preferences, where the lowest score corresponded to ‘strongly disagree’ and the highest score corresponded to ‘strongly agree.’ For the comparative evaluation, we used trend indicators and scales based on means and standard deviations calculated for each scale of the question. To identify and rank respondents’ perceptions regarding knowledge, attitudes, and practices towards COVID-19, we used weighted indices to evaluate questions with different measurement scales. The weighted indices are expressed as percentages. When comparing the average values obtained for two or more responses to which the evaluation scale was different, the value of the percentage index indicating the representativeness of the response as well as the average level of agreement or disagreement for each question is presented.

For the second stage of analysis, an MCDA tool was developed in MS Excel, with spreadsheets containing all criteria, the performance categories corresponding to each criterion, and their scoring functions. The identified criteria were incorporated into a Multi-Criteria Decision Analysis (MCDA) framework, with weights assigned based on their relative importance using the SMART (Simple Multi-Attribute Rating Technique) method, originally developed by Edwards in 1971 (Patel et al. 2017) [24]. This technique involves ranking criteria by importance, assigning scores from 10 (least important) to 100 (most important), and normalizing the total to determine their respective weights. Weighting methods can be broadly classified into subjective and objective approaches. Subjective methods rely on decision-makers’ judgments, which, while intuitive, can introduce bias and overlook objective data. In contrast, objective methods—such as entropy, mean weight, and standard deviation—use mathematical calculations to determine weights, minimizing human influence and ensuring a more data-driven approach [25]. In this study, the criteria were weighted based on the ranking of means and standard deviations derived from respondents’ perceptions in the first stage of data collection. These weighted criteria were then grouped into four dimensions according to their relative importance, with assigned weights expressed as percentages (ranging from 10% to 100%). Each criterion and alternative were assigned weight coefficients that were integrated into the multi-criteria analysis (risk assessments and scenario simulation). The criteria for each dimension were classified into attributes and quantified. The value of each attribute of a criterion was transferred to the total ranked column, and the total weighted value of the criterion was calculated by summing the value calculated for each criterion [6,24]. Based on the linear function U_j_ = Σ_k_ × w_k_ × u_jk_ (where U_j_ represents the utility value of alternative j for the corresponding criterion, w_k_ is the normalized weight of criterion k, and the value u_jk_ is the scaled value of alternative j for criterion k), we calculated the maximum utility value of each alternative identified for each assigned criterion. Then, the alternative with the highest score (ΣU_j_-maximum performance/utility) was considered the optimal alternative. To allow for normalization of the relative importance in weights, a coefficient weight of the requirement for each dimension (criterion), w_k_, was considered, the total of which did not exceed the value of one unit (1.00) [26].

Thus, the essential criteria identified based on the perspectives and preferences of the respondents were weighted, ranked, and evaluated in order of importance, and the alternative with the highest score was considered the best option.

The research also has some limitations, including potential cognitive biases and possible reporting errors in the self-reported data, the lack of external validation of the model, and the lack of sensitive scenario analysis since we did not use MCDA software. A key factor affecting representativeness is selection bias, as the responses were collected from a specific sample of professionals whose experiences and perspectives may not fully generalize to the entire health system. In addition, response bias, such as the tendency to provide socially desirable answers or exaggerate certain problems, may influence the distribution of the analyzed perceptions. The weighting of the questionnaire responses had the objective of decreasing the subjectivity of the response and implicitly the relevance of the response, which together led to the reduction of the bias elements associated with a standard questionnaire.

## 3. Results

In order to comply with the design of the study and the main objectives of our research, the results are presented accordingly: (1) Identifying the essential criteria based on the perceptions of the respondents; and (2) evaluating the identified criteria to find the best alternative option to improve the national health system, aiming to answer the key research question: “*Could MCDA, using a linear mathematical approach that incorporates the qualitative and care needs of patients, serve as a model for decision-makers in improving the resilience of health systems?*”.

### 3.1. First Stage of Analysis: Identifying the Essential Criteria for Enhancing the Healthcare System Based on Knowledge, Attitudes, Preferences, and Perceptions of Respondents

This analysis focused on identifying the criteria which are necessary to improve the national healthcare system through analyzing the attitudes, knowledge, preferences, and perceptions of the responders according to the obtained scales (means and standard deviation for each scale), weighting indices (expressed as percentages), and trend indicators, following the structure of the study in line with the specific objectives proposed:

- Identifying factors that could influence the practice of healthcare professionals, as well as the possible solutions/opportunities that should be implemented in the future to deal with similar situations.
- Identifying alternative solutions to optimize patient accessibility to healthcare services, as well as identifying the risks encountered by patients in accessing healthcare services during a state of emergency and alert.
- Identifying the barriers/limits and possible solutions (risks and opportunities) offered by healthcare professionals for a resilient healthcare system.

#### 3.1.1. Sociodemographic and Behavioral Characteristics

We first collected the demographic characteristics of the respondents and their level of knowledge and expertise in the field and, through assigning weights (as described in Section 2.5), the answers provided by the participants could be more easily sorted in the sections that aimed to assess their knowledge, attitudes, and preferences towards COVID-19, as well as possible alternative solutions and measures.

A total of 412 participants filled in the questionnaire, achieving a response rate of 93.20% (384/412). Among the respondents, 79.85% were doctors, 10.43% were nurses, and 9.22% were pharmacists. The mean rank age was 40.77 ± (SD) 8.43 years old, and more than half were female (65.04%). Around 79% (79.36%) of participants had a higher level of knowledge and education (medical degrees, Ph.D., academic titles) and almost 55% (54.85%) healthcare professionals worked in the civil and private sector in Romania, while 45.15% worked in the National Public Order and National Security System (Table 1).

#### 3.1.2. Knowledge, Attitude, and Preferences of the Respondents Towards COVID-19

Based on the sociodemographic characteristics and behaviors identified from the responses provided by the participants and the questions using measurement scales to assess the new situations generated by the COVID-19 pandemic, according to the process described in Section 2.5, we aimed to identify the factors that could influence the practice of healthcare professionals, as well as possible solutions/opportunities that should be implemented in the future to deal with similar situations.

##### 3.1.2.1. Respondents’ Knowledge, Attitudes, and Preferences When Dealing with New Professional Situations Generated by the COVID-19 Pandemic

To better assess the responses provided regarding respondents’ attitudes and preferences towards COVID-19, we aimed to determine the percentage of healthcare professionals who were involved in anti-pandemic missions and who had already used telemedicine as an alternative service in their practice. From the sociodemographic characteristics and behaviors section, we observed that 24% of respondents stated that they were not involved in any intervention or activity to prevent the spread of SARS-CoV-2 infection and only 8% of participants did not provide an answer, most likely due to non-involvement. The remaining participants (68%) stated that they were actively involved in specific missions. Of these, 25% were involved in the vaccination campaign and epidemiological triage centers, 20% were assigned to COVID-19 support hospitals, 9% were assigned to civilian hospitals, and 14% of participants were involved in case monitoring and surveillance activities. Regarding the use of telemedicine, 27.42% of respondents did not provide an answer (probably due to non-use of this type of service) while 72.58% stated that they had used this alternative service. Of those that already used telemedicine, 32.28% considered it useful only for certain types of consultations, 26.41% considered it useful only for certain categories of patients, and 14.31% chose it as a solution that should be made permanent (Table 1).

##### 3.1.2.2. Respondents’ Perceptions Regarding the Factors That Could Influence Good Practice in Dealing with Crisis Situations

To identify and rank respondents’ perceptions of the factors that could influence the practice of healthcare professionals, we used weighted indices to evaluate questions with different measurement scales, as described in Section 2.5. Based on the comparative evaluation of questions with different measurement scales using weighted indices and weighted averages, we were able to assess that the increased risk of infection, workload, and overload contributed equally to the overload felt by medical staff during the COVID-19 pandemic (both state of emergency and alert) having a similar weighted index rank (15.77), with over 50% of the analyzed responses having a mean rank of 15.67 and an SD of ±0.19 (Table 2).

**Figure 1:**
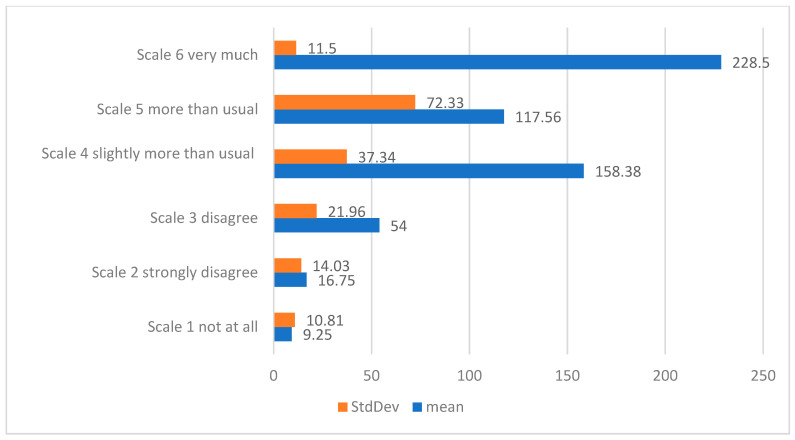
The scale distribution of the mean and standard deviation of the answers to the questions regarding the degree of overload that the medical and sanitary personnel felt during the state of emergency and alert.

After evaluating the distribution of means and standard deviations for each scale of the question in the section, we observed that the most common factor perceived by participants was the degree of overload due to the high volume of tasks and the pressure exerted by the increased risk of infection, highlighted by the trend in the responses, with most participants selecting the maximum value of 6 points in the Likert scale (IQR 6.0–6.0; scale 1 [not at all] to 6 [very much]), with a mean rank of 228.5 (SD ±11); see Figure 1.

Although the assessment of the degree of overload may vary from one individual to another and is influenced by several factors, we can appreciate, from the responses provided by the participants, that the most frequent causes were represented by:

- Increased risk of infection, represented by respondents selecting the maximum value (Likert scale 6, IQR 6.0–6.0; scale 1 [not at all] to 6 [very much], with a weighted rank index of 15.7), followed in the ranking by
- High workload, represented by respondents selecting a value of 5 on the Likert scale (IQR 5.0–6.0; scale 1 [not at all] to 5 [a little more than usual], weighted rank index of 15.77), and
- Lack of resources as well as new professional situations generated by the COVID-19 pandemic, represented by a value of 4 in the responses (Likert scale 4, IQR 4.0–6.0, scale 1 [not at all] to 4 [approximately as much as I had in the state of alert], weighted index of 15.4).

##### 3.1.2.3. Responders’ Perceptions Regarding Practice and Preferences to Increase Patient Accessibility to Healthcare Services

**Figure 2:**
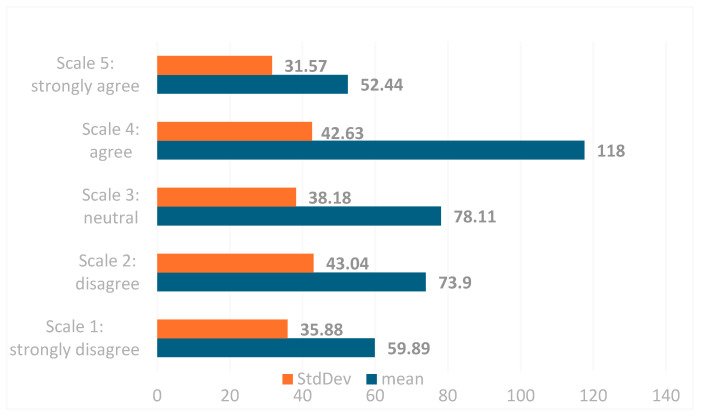
The scale distribution of the mean and standard deviation of the answers to the questions regarding the impacts on patients’ access to medical services during the state of emergency and alert.

Evaluating the trend in responses regarding patients’ access to health services during the COVID-19 pandemic, the general perception of the majority of respondents was represented by the need to develop and implement alternative solutions, such as the use of telemedicine services, to increase the accessibility of vulnerable groups of patients to health services—a fact demonstrated by their selecting a value of 4 on the Likert point scale (IQR 4.0–5.0, scale 1 [not at all] to 5 [very much], with a mean of 118 and a standard deviation of ±42.63), representing the agreement of healthcare professionals based on patients’ needs regarding the use of telemedicine (Figure 2).

Based on the comparative evaluation of questions with different measurement scales and the resulting weighted indices and weighted averages, we can conclude that the highest values revealed that the access of vulnerable groups to medical services was lacking, represented by 47.12% of respondents choosing the maximum value (Likert scale 5, IQR 5.0–5.0, scale 1 [totally disagree] to 5 [totally agree], with a weighted index of 15.05). Furthermore, 14.94% of respondents agreed that the number of consultations was affected, choosing a value of 4 on the Likert scale (IQR 4.0–5.0, scale 1 [totally disagree] to 4 [agree], with a weighted index rank of 14.94; see Table 3).

At the same time, due to the temporary closure of some health facilities and the low number of consultations, 37.34% of the participants reported choosing to use telemedicine services as an alternative option to increase the access of patients to healthcare services (Likert scale 5, IQR 5.0–5.0, scale 1 [strongly disagree] to 5 [strongly agree], weight index rank 14.74). Due to the underfunding of the medical system, even the incomes of the medical personnel were affected, and 35.66% of the participants selected the Likert point scale 3 in this context (IQR 3.0–5.0, scale 1 [strongly disagree] to 3 [neutral], weight rank index of 14.1), with this answer being third in the hierarchy in terms of its influence on the accessibility of patients (Table 3).

According to the respondents’ perception, the main factors that led to the decrease in patients’ access to medical services during the pandemic (both in the state of emergency and alert) were represented by:

- Lack or low access to medical services for patients belonging to vulnerable groups, a fact represented by the strong agreement given by the respondents by selecting the maximum (Likert scale 5, IQR 5.0–5.0, scale 1 [totally disagree] to 5 [totally agree], weight rank index of 15.05), followed by
- Underfinancing of the medical system and temporary closure of some health facilities, a fact represented by respondents selecting a value of 3 on the Likert scale (IQR 3.0–5.0, scale 1 [totally disagree] to 3 [neutral], weight rank index of 14.1), and
- Lack of preparation of patients in the use of telemedicine as an alternative service, a fact represented by the agreement given by respondents through selecting a value of 4 on the Likert scale (IQR 4.0–5.0, scale 1 [totally disagree] to 4 [agree], weight rank index of 11.61; see Table 3).

#### 3.1.3. Respondents’ Perceptions Regarding the Risks (Barriers/Limits) and Opportunities (Solutions) for a Resilient Healthcare System

Based on the same algorithm that we used in the previous sections, we were able to assess that the increased risk of infection, poor communication, lack of financial support for the medical system, patient reluctance, the lack of a harmonized legal framework, and overload contributed equally to the decrease in the resilience of medical personnel in Romania during the COVID-19 pandemic (in both emergency and alert states), a fact represented by their selecting the maximum value on the Likert scale for all identified factors and the similar values obtained for the weighted indices.

The solutions offered by respondents in the qualitative and feedback sections, based on sociodemographic and behavioral characteristics (Table 1), are closely connected to the limits/barriers identified following the assessment of respondents’ perceptions regarding the main factors that led to the decrease in the resilience of the health system during the pandemic (Table 4), as summarized below:

- The need to establish preventive measures, with 34% of respondents offering the establishment of educational, screening, and vaccination campaigns as solutions due to the high risk of infections, with an index weight of 73.94% indicating the representativeness of the response, as well as the average level of agreement represented by their selecting the maximum value of 5 on the Likert scale (IQR 5.0–5.0, scale 1 [totally disagree] to 5 [totally agree]);
- Improving communication between authorities/decision-makers and healthcare professionals (29% of respondents reported poor communication from the authorities, as well as dysfunctions of the medical system), with an index weight of 85.45% indicating the representativeness of the response, as well as the average level of agreement represented by their selecting the maximum value of 5 on the Likert scale (IQR 5.0–5.0, scale 1 [totally disagree] to 5 [totally agree]);
- The need to finance the system (12% of respondents reported underfinancing of the medical system) is naturally related to the underfunding of the system, represented by an index weight of 41.14% which indicates the representativeness of the response, as well as the average level of agreement represented by their selection of the maximum value of 5 on the Likert scale (IQR 5.0–5.0, scale 1 [totally disagree] to 5 [totally agree]);
- The need to establish a legal harmonization framework, represented by an index weight of 52.02% indicating the representativeness of the response, as well as the average level of agreement represented by their selection of the maximum value of 5 on the Likert scale (IQR 5.0–5.0, scale 1 [totally disagree] to 5 [totally agree]).

**Table 4:** The distribution of the weight index value/percentage (%) and scales, according to respondents’ perceptions regarding the risks (barrier/limits) for enhancing the healthcare system.

Questions	Risk Factors	Weight Index Value and %		Preferred Response
Q3.1.	High risk of infections	15.58	73.94	5
Q3.3	Poor communication	15.67	84.45	5
Q3.4	Underfunded system	15.44	41.14	5
Q3.5	Reluctance of the patients	15.77	56.47	2
Q3.8	Lack of a harmonized legal framework	15.02	52.02	5

### 3.2. Second Stage of Analysis: Evaluation of the Identified Criteria for Determination of the Best Alternative Option to Enhance the National Healthcare System

Based on the findings resulting from the first stage of analysis, we conducted a risk assessment of the identified criteria and proposed three simulation scenarios (alternatives/strategies) to evaluate possible measures adopted in the short-, medium-, and long-term to improve the national healthcare system. The alternatives/strategies (S_n,n=1,2,3_) were weighted with the same coefficient weight (w_k_ = 0.35) and ranked (r_sn,n=1,2,3_) according to the importance of the outcomes of possible actions (w_ksn,n=1,2,3_); see Table 5.

The risks and solutions (key attributes, A_n,n=1,2,3,4_) identified in the first stage of the analysis were grouped into four dimensions (criteria), as follows: K1 (1st dimension)—Human Resource Management; K2 (2nd dimension)—Healthcare service planning; K3 (3rd dimension)—Budget allocation; and K4 (4th dimension)—Legal framework.

The criteria in each dimension were weighted to allow for the normalization of relative importance via weights based on the perceptions and preferences of the respondents, according to the algorithm described in Section 2.5. Therefore, the highest weight was assigned to the third dimension (budget allocation; w_3_ = 0.35), in order to emphasize the maximum importance and necessity of implementing measures regarding the allocation of resources (material, financial, and personnel), given the difficulties encountered by the system during the pandemic. The lowest weight coefficient was assigned to the fourth dimension (Legal framework; w_4_ = 0.15), as it could be influenced by the political factor. Regarding the other two dimensions—Human Resources Management and Healthcare service planning—coefficients of w_1_ = 0.2 and w_2_ = 0.3 were assigned, respectively, given that the identified risks were a lack of trained and qualified personnel, and telemedicine had already been implemented as an alternative solution during the pandemic (Table 6).

We emphasize that scenario modeling involves uncertainties and assumptions [27], which is why we chose MCDA in our analysis, which allowed for an organized and quantitative assessment of alternative criteria. Each dimension was evaluated under each proposed alternative/strategy using the performance matrix and the SMART algorithm. Based on the linear function described in Section 2.5, each alternative was compared according to each identified dimension, and the alternative with the highest score (maximum performance or utility) was considered the optimal alternative.

When ranking by alternatives/strategies, it was ensured that the total was the same (14) and the sum of the weighted coefficients per criterion was equal to 1, as shown below (Table 7).

The matrix for each scenario simulation evaluated the alternatives/strategies and ranked them in order of importance from lowest to highest. Each box indicated the extent to which the corresponding alternative satisfied the given attribute of the dimension (criterion), with the results expressed in weighted terms. The performance matrix relies on a linear additive model, in which the overall value of an alternative is calculated as the sum of the performance score (value) of each criterion (dimension) multiplied by the weight of that dimension (criterion). For each alternative/strategy, the score was calculated and the alternative that obtained the highest score was considered as the optimal alternative [28]. The optimal strategy was option three, having the highest weighted total (3.90), as shown in Table 8, which suggests that this could be the optimal solution for increasing the resilience of the healthcare system; more specifically, there is a need to implement long-term solutions.

The results provided by the linear model described in Section 2.5 were similar to those obtained in the performance matrix, in the sense that alternative 3 (j3)/strategy S_3_, obtained the highest score (U_j_ = 1.14), representing the optimal alternative that should be selected or the alternative with the maximum performance or utility [29] regarding the analyzed objective (Table 9).

The alternative with the highest score (maximum utility value or maximum performance of the alternative) [30] represents the best alternative [31], which in our case was the third alternative/third strategy, more precisely, the long-term implementation of solutions that ensure the sustainability and efficiency of the medical system to prevent similar situations as COVID-19 pandemic.

## 4. Discussion

Our study aimed to determine whether MCDA and the SMART technique can serve as a model for clinicians and decision-makers in selecting the best possible options when faced with complex situations that require prioritization and urgent measures. The findings differ from other research utilizing MCDA in healthcare due to the distinct methodology and objectives we proposed. Specifically, while studies employing the SMART method have primarily focused on selecting the most suitable therapy or technology—often within pharmacoeconomic and cost-effectiveness analyses—our approach aligns with the National Health Strategy 2023–2030, developed by the Ministry of Health. By emphasizing the role of MCDA in healthcare decision-making, our study integrates national strategic objectives and explores potential tools to strengthen the national healthcare system, particularly through the application of the SMART technique as a structured and transparent decision-making tool.

The present work was based on a survey issued to 412 healthcare professionals in order to assess their perceptions, attitudes, and knowledge regarding the responses to pandemic conditions. The research consisted of two stages of analysis where, based on the respondents’ perceptions and with the help of the MCDA and additive linear model, we aimed to achieve the main objectives of the study; that is, to identify the essential criteria (risks and opportunities) and alternative solutions for optimizing and reforming the health system and, subsequently, identifying the most likely strategy/best alternative option for increasing the resilience of the national health system.

The findings from the analysis of the first stage were structured according to the study design. Thus, from the first and third sections of the first stage of analysis, based on the sociodemographic behavioral characteristics and the open-ended responses in the qualitative questionnaire and the feedback section, we gathered valuable data on the behavioral traits of the participants during the COVID-19 pandemic. These included their attitudes, involvement in efforts to combat the virus, use of telemedicine, lessons learned from the pandemic, and opinions on increasing the resilience of the national healthcare system to prevent future crises. From the second section of the first stage, the resulting data showed that, although the perceptions of the respondents may vary considerably from one individual to another, the degree of physical and mental overload of the medical staff was exacerbated by the increased risk of infection, the high workload, the increased demand for medical services, the limited resources, and the unprecedented professional challenges they faced during the pandemic. Furthermore, the temporary closure of certain healthcare facilities and the reduction in the number of consultations highlighted the critical need to explore alternative solutions, with telemedicine being considered a viable means of improving the accessibility of healthcare for all categories of patients. The lack of qualified and trained personnel, as well as the high workload and multiple tasks, insufficient financing of the medical system, the lack of a regulatory framework and protocols, and patient reluctance to adopt control and prevention measures, as well as the decrease in patient access to medical services during the pandemic, were the most common causes perceived by respondents. The solutions offered by the survey participants were closely related to the identified risks, consisting of hiring trained and qualified personnel, better planning of medical services and the use of telemedicine as an alternative service to ensure access to medical services for all patients, financing the system, and creating a harmonized legal framework. Taken together, these perspectives highlight the need to provide continuous support through investments in the development and research of new innovative solutions in the healthcare sector. In the second stage of analysis, all these risk factors and proposed solutions resulting from the first stage were considered when establishing the evaluation criteria and normalizing the values for the analysis matrix, in order to identify the best alternative option/the strategy most likely to increase the resilience of the national healthcare system. Considering the above, the entire process of this work attempted to answer the question of whether MCDA using a linear additive model could serve as a model to support decision-makers in improving the resilience of healthcare systems. Risk assessment and scenario simulation are powerful tools to help make informed policy decisions, helping to minimize the risks associated with managing situations similar to the COVID-19 pandemic. MCDA empowers decision-making groups to make informed and transparent choices, even when faced with extremely complex situations. This method can be used to serve as an index that helps with decision-making during the sorting of alternatives into pre-defined sets [31] (p. 16). The study demonstrated a potential application of the MCDA and SMART methods in finding the best alternative option, in our case the long-term strategy being the best alternative option for increasing the resilience of the national health system.

Our research successfully identified the proposed objectives by developing an empirical example of a tool that can serve as a support for decision-making based on local perspectives and needs. The results produced by MCDA suggest a promising approach for improving preparedness and response efforts, with the significant benefit of allowing for the re-evaluation of strategies or measures adopted. This empirical model may have different results depending on the problem addressed, geographical factors, the inclusion of decision-makers and the use of integrated MCDA software. The applied model has the potential to serve as a valuable decision-making tool for clinicians and policymakers in navigating complex situations that require urgent prioritization and action. Moreover, it aligns with national strategic objectives by exploring mechanisms to strengthen the healthcare system. However, certain limitations must be acknowledged. The absence of an MCDA approach incorporating software-based solutions or advanced methodologies—our study being centered on a structured framework requiring further empirical validation—may impact the model’s practical applicability. Additionally, the limited sample, which included only healthcare professionals and excluded policymakers, may restrict the generalizability of our findings. Future research should explore the integration of AI-driven tools with MCDA to enhance data fidelity, accuracy, and transparency. Expanding the sample to include decision-makers would further improve representativeness and support a more comprehensive validation of the model in real-world applications or comparative analyses with alternative decision-making frameworks. Moreover, selection bias may limit the broader applicability of our findings, as responses were collected from a specific professional group whose perspectives may not fully capture the diversity of the healthcare system. Response bias—such as the tendency toward socially desirable answers or a focus on particular issues—could also influence results. While weighting responses helped mitigate subjectivity, the inherent biases associated with standard questionnaires remain a consideration.

## 5. Conclusions

While Multi-Criteria Decision Analysis (MCDA) is gaining ground in various healthcare decision-making contexts, there is a dearth of publications that provide guidelines and best practices for conducting high-quality research using MCDA. This research underscored the importance of treatment, adherence, and quality of life, contributing to informed, patient-centered decision-making and strengthening the resilience of healthcare systems.

This study also highlighted the importance and benefit of integrating MCDA into the decision-making process to prioritize resources and optimize action plans (thus, mitigating threats), as well as capitalizing on opportunities focused on patient needs and practitioner preferences, promoting engagement and consensus among stakeholders (i.e., practitioners and decision-makers) in line with local perspectives and priorities. The presented approach integrates both qualitative and quantitative data, incorporating patient values and preferences to develop meaningful solutions, particularly when considering crisis situations requiring urgent action. The most common risks and solutions resulting from the knowledge, attitudes and perceptions of respondents during the COVID-19 pandemic (state of emergency and alert) were as follows:

(1) A lack of qualified and trained personnel, a risk identified by the high degree of physical and mental overload perceived by respondents due to the high volume of tasks and the pressure exerted by the increased risk of infection, as well as the new professional situations they faced (see Section 3.1.2.1 Respondents’ Knowledge, Attitudes, and Preferences When Dealing with New Professional Situations Generated by the COVID-19 Pandemic).
(2) Poor communication from the authorities, as well as the dysfunctions of the medical system leading to decreased access to medical services by patients, a risk perceived by respondents in Section 3.1.2.2 Respondents’ Perceptions Regarding the Factors That Could Influence Good Practice in Dealing with Crisis Situations and Section 3.1.3 Respondents’ Perceptions Regarding the Risks (Barriers/Limits) and Opportunities (Solutions) for a Resilient Healthcare System, led to the recommendation of the need to develop and implement cost-effective alternative solutions to ensure proper planning and organization of medical services, with telemedicine being identified as a viable option (see Section 3.1.1 Sociodemographic and Behavioral Characteristics and Section 3.1.3 Respondents’ Perceptions Regarding the Risks (Barriers/Limits) and Opportunities (Solutions) for a Resilient Healthcare System).
(3) The inability of the health system to effectively manage crisis situations, such as reluctance to implement control and monitoring measures, a risk identified based on the perceptions of respondents discussed in Section 3.1.3 Respondents’ Perceptions Regarding the Risks (Barriers/Limits) and Opportunities (Solutions) for a Resilient Healthcare System and the solutions offered by them that included adequate financial allocations for the financing of the system (see Section 3.1.1 Sociodemographic and Behavioral Characteristics).
(4) The lack of regulatory oversight identified based on the perceptions in Section 3.1.3 Respondents’ Perceptions Regarding the Risks (Barriers/Limits) and Opportunities (Solutions) for a Resilient Healthcare System, led to the recommendation of the need to establish a harmonized legal framework that ensures a coordinated and unitary response from the authorities and, at the same time, allows for monitoring of compliance with the implemented norms and measures (see Section 3.1.1. Sociodemographic and Behavioral Characteristics and Section 3.1.3 Respondents’ Perceptions Regarding the Risks (Barriers/Limits) and Opportunities (Solutions) for a Resilient Healthcare System). The SMART was successfully applied to determine the ranking and weighting of the criteria in order of importance (beginning with the least favorable levels of the criteria and working up to the most favorable) and the alternative with the highest score was considered the best alternative option that should be adopted considering more than one criterion in the selection process.

The findings from the second stage of analysis indicated that the Romanian healthcare system requires a long-term strategy for the implementation of measures to enhance its resilience, including adequate financing, efficient planning and organization of healthcare services, adequate management of healthcare personnel, and the establishment of a cohesive legal framework. Considering all these points, we strongly believe that adopting MCDA and a linear mathematical approach that incorporates quantitative and qualitative data focused on patients’ needs and healthcare experts’ preferences could serve as a model to support decision-makers in improving the resilience of healthcare systems.

The applied example could serve as a potential decision-making tool for clinicians and decision-makers in complex situations requiring urgent prioritization and action and also aligns with national strategic objectives to explore potentials tools for strengthening the national health care system.

The lack of an MCDA approach that integrates software-based solutions or advanced methodologies (our study focused on a structured framework that requires further empirical testing) the sample size (we excluded decision-makers) and possible errors and bias are possible limitations in validating the model. The next steps for future studies could involve the following directions to enhance and expand the methodology:

(1) Dedicated Software Integration: While our study employed Excel formulas with respondent-derived weights, developing a dedicated software solution could automate the process and allow integration with real-time data. This would not only improve precision but also support dynamic decision-making in urgent clinical scenarios.
(2) Refinement of Weighting Criteria: Future research could involve a broader and more diverse pool of clinicians to fine-tune the weight assignments. This would help in capturing a wider range of perspectives, thus, enhancing the robustness of the decision model across various healthcare settings.
(3) Bias Reduction and Error Minimization: Implementing advanced statistical techniques and sensitivity analyses may help address potential reporting errors and biases. This step would further solidify the reliability of the method, ensuring that the outcomes remain consistent under different conditions.
(4) Integration with Clinical Decision Support Systems (CDSS): Exploring the integration of our MCDA-SMART approach within existing CDSS could facilitate broader adoption. This integration could provide clinicians with a seamless, user-friendly tool that supports evidence-based decision-making at the point of care.
(5) Expanded Clinical Trials and Simulation Studies: Conducting multi-center studies or simulation trials in diverse healthcare environments will be crucial to validate the method’s effectiveness and scalability. These studies could offer insights into the adaptability of the approach and identify any context-specific adjustments needed.

By following these directions, we aim to improve the utility and accuracy of our approach, ultimately supporting clinicians to make more informed decisions in complex and time-sensitive scenarios, and we also hope to inspire future health research and the development of novel strategies to navigate complex clinical scenarios.

## Figures and Tables

**Table 1:** Demographic and behavioral characteristics of the participants (n = 412).

Variable	No. (%)
**Age, mean ± SD**	40.77 ± (SD) 8.43
**Sex**	
Male	144 (34.95%)
Female	268 (65.04%)
**Education**	
Post-university	327 (79.36%)
University	36 (8.73%)
Pre-university	49 (11.89%)
**Healthcare profession**	
Doctors	329 (79.85%)
Pharmacists	38 (8.73%)
Medical assistants	49 (11.89%)
**Medical system**	
National Private Sector	57 (13.83%)
National Civil Sector	169 (41.02%)
National Public Order and National Security System	186 (45.15%)
**Involvement in COVID-19 pandemic**	
Not involved	99 (24%)
Did not provide an answer	33 (8%)
Vaccination campaign/epidemiological triage centers	103 (25%)
COVID-19 military support hospitals	82 (20%)
Activities of monitoring and supervising cases (DSPs, ONGs)	58 (14%)
Civil hospitals	37 (9%)
**Using telemedicine**	
Alternative solution only for some type of consultation	133 (32.28%)
Alternative solution for some categories of patients	109 (26.41%)
Alternative solution only for the pandemic state	74 (17.85%)
Alternative solution that should be permanent	59 (14.46%)
Other situation/missing the answer	37 (9%)
**Telemedicine limits and barriers**	
The difficulty of examination	140 (34%)
The difficulty of establishing a diagnosis	79 (19.17%)
The patients are not instructed	47 (11.29%)
Technical issue–internet network	17 (4.12%)
Another situation	16 (4%)
Not providing an answer	113 (27.42)
**Perceptions/solutions/recommendations**	
The need to institute preventive measures like screening, vaccination campaigns, educational campaigns	140 (34%)
Poor communication from the authorities and malfunctions of the medical system	119 (29%)
Underfunding of the medical system	49 (12%)
The need for developing new solutions for monitoring and supervising the patients	17 (4%)
I don’t know/other situations	87 (21%)

**Table 2:** Perception of the respondents regarding the factors that could influence the practice of healthcare professionals in crisis situations based on weighted indices and scales.

Questions	Risk Factors	Weight Index Value and %		Preferred Response
Q1.1.	Mental pressure because of the high risk of infection	15.77	39.92	4
Q1.2	Physical overwork generated by wearing protective equipment	15.4	47.92	4
Q1.3	Different workload differentiated by specialties	15.53	54.82	5
Q1.4	Workload in state of emergency	15.77	58.77	6
Q1.5	Workload in state of alert pandemic	15.77	53.56	6
Q1.6	Different tasks and workload in state of emergency pandemic	15.64	58.52	5
Q.1.7	Different tasks and workload in state of alert pandemic	15.69	56.71	5
Q1.12	Physical and mental overwork	15.77	38.31	4

**Table 3:** The distribution of answers according to the weighting indices and the scales selected by the respondents, regarding the accessibility of patients to medical services during the state of emergency and alert.

Questions	Risk Factors	Weight Index Value and %		Preferred Response
Q2.1	The total number of consultations affected by the spread of the virus	14.94	42.15	4
Q2.2	Income impact	14.1	35.66	3
Q2.3	The consultations of the vulnerable group of patients affected	15.05	47.12	5
Q2.4	Using telemedicine as an alternative service	14.74	37.34	5
Q2.5	The lack of instruction for the patients in using telemedicine service	11.61	48.92	4
Q2.6	Overall satisfaction of the medical personnel using the telemedicine	12.13	38.59	4
Q2.7	Overall satisfaction of the patients using telemedicine	10.82	43.5	4
Q2.8	Overall satisfaction regarding telemedicine compared to classic consultations	12.74	38.43	3
Q2.10	Need for instructions/training for using telemedicine from the patients	14.61	61.9	3

**Table 5:** Ranking the alternatives according to the relative importance of the outcomes of possible actions.

Description of the Alternatives (Strategies) (S_n,n=1,2,3_)	Ranking Importance of Criterion Per Alternative/Strategy (r_s1,_ r_s2_, r_s3_),	Weight Coefficients (w_k_)	Total Ranking of Alternative (w_ks1_,w_ks2_,w_ks3_)
**S1: prioritization in emergency regime** Focuses on quick actions to mitigate risks or pressing issues; it may not provide a long-term solution or might overlook systemic changes for sustainability.	1	0.35	0.35
**S2: medium-term solutions** Emphasizes planning and resource allocation over a medium timeframe. It allows for adjustments based on emerging data and trends, making it a step towards resilience.	2	0.35	0.70
**S3: long-term prioritization** A comprehensive approach that includes sustainable funding, effective service planning, and legal frameworks. It reflects a commitment to long-term improvements, which is vital for building a robust healthcare system capable of handling future crises.	3	0.35	1.05

**Table 6:** Ranking and rating the criteria classified in the four considered dimensions based on their relative importance.

Description of the Criterion k (Dimensions)	Criteria/Key Attributes (Risks/Opportunities) (A_n,n=1,2,3…_)	Normalization of the Relative Importance of the Dimension into Weights (w_1,2,3,4_)
**K1** (1st dimension) **Human Resource Management** Ensuring staff are well-managed, supported, and trained is fundamental to maintaining a responsive healthcare system.	A.1. The shortage of trained and qualified personnel	0.20
**K2** (2nd dimension) **Healthcare service planning** Long-term planning for healthcare services can help to anticipate and address future challenges, ensuring that the system is prepared for various scenarios.	A.2.1. Solutions for proper planning and organization of medical services	0.30
	A.2.2. Educational and informing campaign for population	
	A.2.3. Ensuring access to medical services for all the patients including those from vulnerable groups	
	A.2.4. Developing cost-effective alternative solutions, like telemedicine	
**K3** (3rd dimension) **Budget allocation** Effective budget allocation is crucial for ensuring that funds are directed towards areas that will yield the highest impact on healthcare delivery and resilience.	A.3. Health system underfunding	0.35
**K4** (4th dimension) **Legal framework** Establishing a solid legal framework can facilitate quicker responses to public health emergencies and ensure accountability and governance in healthcare delivery.	A.4. Lack of a harmonized legislation	0.15
**Total value weights assigned** (Σ_k_ × w_k_ = 1)		**1**

**Table 7:** (1) Risk assessment and scenario simulations for the first alternative/strategy: Prioritization in emergency regime. (2) Risk assessment and scenario simulations for the second alternative/strategy: Medium-term solutions. (3) Risk assessment and scenario simulations for the third alternative/strategy: Long-term prioritization.

**(1)**					
**Attribute of** **Dimension/** **Criterion** **(A/K)**	**Ranking** **Importance of Attribute of Dimensions/** **Criterion**	**Normalization of the Relative Importance of the Dimension into Weights** **(w_1,2,3,4_)**	**Ranking** **Weights** **Assigned per Attribute of** **Dimension** **(u_j1k1_)**		**Ranking** **Weights** **Coefficient/Variant 1 Based on Dimension**
A1/K1	1	0.2	0.2		0.2
A2.1/K2	3	0.3	0.9		0.27
A2.2/K2	1		0.3		
A2.3/K2	3		0.9		
A2.4/K2	2		0.6		
A3/K3	1	0.35	0.35		0.35
A4/K4	3	0.15	0.45		0.45
Total value weights assigned for S1	14	1	3.7		3.7
**(2)**					
**Attribute of** **Dimension** **(A/K)**	**Ranking** **Importance of Dimension**	**Normalization of the Relative Importance of the Dimension Into Weights** **(w_1,2,3,4_)**	**Ranking** **Weights** **Assigned per Attribute of** **Dimension(u_j2k2_)**	**Ranking** **Weights** **Coefficient/Variant 2 Based on Dimension**	
A1/K1	2	0.35	0.7	0.7	
A2.1/K2	2	0.3	0.6	2.4	
A2.2/K2	2		0.6		
A2.3/K2	1		0.3		
A2.4/K2	3		0.9		
A3/K3	2	0.2	0.4	0.4	
A4/K4	2	0.15	0.3	0.3	
Total value weights assigned for S2	**14**	**1**	**3.80**	3.80	
**(3)**					
**Attribute of** **Dimension** **(A/K)**	**Ranking** **Importance of Dimension**	**Normalization of the Relative Importance of the Dimension Into Weights** **(w_1,2,3,4_)**	**Ranking** **weights** **Assigned per Attribute of** **Dimension** **(u_j3k3_)**	**Ranking** **Weights** **Coefficient/Variant 3 Based on Dimension**	
A1/K1	3	0.35	1.05	1.05	
A2.1/K2	1	0.3	0.30	2.1	
A2.2/K2	3		0.30		
A2.3/K2	2		0.60		
A2.4/K2	1		0.30		
A3/K3	3	0.2	0,60	0.60	
A4/K4	1	0.15	0.15	0.15	
Total value weights assigned for S3	**14**	**1**	**3.90**	3.90	

**Table 8:** The performance matrix.

Dimension/Criterion (K)	Weight Coefficients (w_k1_, w_k2_, w_k3_)	Ranking Weights Coefficients Assigned for 1st Alternative (S1)	Ranking Weights Coefficients Assigned for 2nd Alternative (S2)	Ranking Weights Coefficients Assigned for 3rd Alternative (S3)
K1 Budget allocation	0.35	0.35	0.7	1.05
K2 Healthcare service planning	0.3	2.70	2.4	2.1
K3 Human Resource Management	0.2	0.20	0.4	0.6
K4 Legal Framework	0.15	0.45	0.3	0.15
Total value weights of alternative	1	3.70	3.80	3.90

**Table 9:** Calculating the utility of the alternative and deciding the alternative with the highest score according to SMART.

Alternative (Strategy) (j)	Ranking Weights Coefficient per Each Alternative (w_k1_,_2,3_)	Ranking Weights Coefficient/k1 (u_j1k1_)	Ranking Weights Coefficient/k2 (u_jk2_)	Ranking Weights Coefficient/k3 (u_jk3_)	Ranking Weights Coefficient/k4 (u_jk4_)	Global Scoring Utility Value for Each Variant U_j_ = Σ_k_ (w_k_ × u_jj_)
S1 (j1)	0.35	0.35	2.70	0.20	0.45	1.04
S2 (j2)	0.70	0.70	2.40	0.40	0.30	1.09
S3 (j3)	1.50	1.05	2.10	0.60	0.15	1.14

## Data Availability

The database used and analyzed during the current study is available from the corresponding author on reasonable request.

## Supplementary Materials

The following supporting information can be downloaded at: https://www.mdpi.com/article/10.3390/jcm14061943/s1, File S1. Effects felt by healthcare professionals in Romania during the COVID-19 pandemic (both emergency and alert).
