# Supplementary material for: SMART Multi-Criteria Decision Analysis (MCDA)—One of the Keys to Future Pandemic Strategies
Source: J Clin Med. 2025 Mar 13;14(6):1943. doi: 10.3390/jcm14061943 (PMC11943005; doi:10.3390/jcm14061943)
Supplement: Supplementary file 1 [file jcm-14-01943-s001.zip › jcm-3486023-supplementary.pdf]

APPLIED QUESTIONNAIRE
"Effects felt by healthcare professionals in Romania during the COVID-19 pandemic (both emergency and alert)"

Please support me by answering the questions in this questionnaire to complete a doctoral research topic on the impact of the COVID-19 pandemic among healthcare personnel.

We assure you that your answers will remain confidential, and the information obtained will be used exclusively for scientific purposes.
We also assure you that the research approach fully complies with the legal provisions regarding the confidentiality of personal data (GDPR). Your identification data (IP/email) are not processed or stored.
Participation in the questionnaire is completely voluntary and anonymous, and is therefore not subject to the GDPR.
The decision to participate or not in this questionnaire will not interfere in any way with your rights as a healthcare professional.
By filling out the form you will give your consent for participation; it takes 3-5 minutes to complete the questionnaire.
Thank you!

SECTION 1: Identifying the degree of overwork of medical personnel in the military system
Hypothesis: The higher the degree of overload of doctors, the lower the efficiency of the medical act.

1. To what extent has the knowledge that any patient is potentially infected with the SARS-COV-2 coronavirus increased stress related to professional activity?
Only one answer
a) To a very large extent
b) To a large extent
c) To a small extent
d) Not at all
e) I don't know/I don't answer

2. To what extent does wearing full protective equipment increase fatigue?
Only one answer
a) To a very large extent
b) To a large extent
d) To a small extent
e) Not at all
f) I don't know/I don't answer

3) So far, during the pandemic, do you consider that there have been differences in workload between medical specialties?
Only one answer
a) To a very large extent
b) To a large extent
d) To a small extent
e) Not at all
f) I don't know/I don't answer

4) Overall, how do you evaluate the workload you had during the state of emergency?
Only one answer
a) Much more than the usual norm
b) A little more than the usual norm
c) About the same as we had before the start of the pandemic
d) Less than the usual norm
e) Much less than the usual norm
f) I don't know / I don't answer

5) Overall, how do you evaluate the workload you have during this period (wave 4)?
Only one answer
a) Much more than the usual norm
b) A little more than the usual norm
c) About the same as we had before the start of the pandemic
d) Less than the usual norm
e) Much less than the usual norm
f) I don't know / I don't answer

6) Regardless of the actual workload, do you consider that the work tasks received during the state of emergency were:
Only one answer
a) More demanding than the usual norm
b) As demanding as during the state of alert
c) Less demanding than the usual norm
d) Much less demanding than the usual norm
e) I don't know / I don't answer

7) Regardless of the actual workload, do you consider that the work tasks received during this period (wave 4) were/are:
Only one answer
a) More demanding than the usual norm
b) As demanding as during the state of emergency
c) Less demanding than the usual norm
d) Much less demanding than the usual norm
e) I don't know / I don't answer

8) Are you or have you been involved in pandemic support actions (combating/preventing COVID-19)?
Only one answer
a) Yes
b) No

**** 9) If you have been/are involved in pandemic support actions, where have you been/are you involved? (COVID-19 support hospital, DSPs, epidemiological triage missions, etc....):***
Arguments
____

**** 10) Can you name at least one new professional situation you have encountered since the beginning of the pandemic?***
Arguments
____

**** 11) Propose solutions that could reduce overload in a similar future situation***
Arguments
____

12) To what extent did you feel overworked (physically and mentally) as a result of the COVID-19 combat/prevention missions carried out?
Scale from 1 to 5, where 1- means not at all and 5 – to a very great extent
a) To a very large extent
b) To a large extent
d) To a small extent
e) Not at all
f) I don't know/I don't answer

*NB *not measured - part of the qualitative section*

SECTION II

Objective: Identifying barriers to patient access to medical services from the doctor's perspective and possible solutions to overcome/prevent them

Hypothesis: If patients with a history of chronic diseases do not have direct access to consultations, then the degree of addressability of patients decreases

1) ***To what extent was the number of patients consulted affected due to the distancing measures that had to be respected?***

Only one answer
a) To a very large extent
b) To a large extent
c) To a small extent
d) Not at all
f) I don't know/I don't answer

2) ***To what extent have revenues been affected by the lower number of patient consultations and higher expenses for the supply of protective materials and disinfectants?***

Only one answer
a) To a very large extent
b) To a large extent
c) To a small extent
d) Not at all
f) I don't know/I don't answer

3) ***During the COVID-19 pandemic, has the care of patients with other conditions been/is being put on the back burner?***

Only one answer
a) Totally disagree
b) Partial disagreement
c) Partially agree
d) Totally agree
f) I don't know/I don't answer

4) ***During the COVID-19 pandemic, have you practiced remote consultations - telemedicine (smartphone , laptop, PC)?***

Only one answer
a) Yes, only during the state of emergency
b) Yes, throughout the pandemic
c)sometimes/another situation
d) Not at all
e) I don't know / I don't answer

5) ***To what extent has your patient become familiar with "telemedicine" (telephone consultations, online laptop/PC)?***

Only one answer
a) Largely all patients who could not move
b) To some extent, only patients are familiar with technology
c) To some extent, only young patients
d) Almost no one
e) I don't know/I don't answer

6) ***Was/is the medical care provided to patients under telemedicine conditions satisfactory to you?***
Only one answer
a) Yes, to a large extent
b) Yes, to some extent
c) Yes, to a small extent
d) No, not at all
e) I don't know/I don't answer.

7) ***Was/is the medical care provided to patients under telemedicine conditions satisfactory for the patient?***
Only one answer
a) Yes, to a large extent
b) Yes, to some extent
c) Yes, to a small extent
d) No, not at all
e) I don't know/I don't answer.

8) ***The quality of consultation and care through "telemedicine" compared to classic consultation is:***
Only one answer
a) Better
b) Almost as good
c) Not very good
d) Almost not good at all
e) Not good at all
f) I don't know / I don't answer

9) ***Which of the following situations represents the main impediment to "telemedicine" consultation?*** *Multiple answer*
a) Internet/telephone connection
b) Difficulty examining the patient
c) Difficulty in establishing the diagnosis
d) Legal impediments
e) Patients are unable/do not know how to use the technique
f) Another situation
g) I don't know/I don't answer.

10) ***Have you received a training course in the field of remote consultations/telemedicine?***
Only one answer
a) Yes, I have benefited from a course/series of courses covering situational variety
b) Yes, I have received training containing basic elements
c) No, I have not received any training regarding remote consultations (telemedicine)
*d) other situation*____
e) Not applicable
f) I don't know / I don't answer

10) Overall, for your "telemedicine" represents a solution:
Multiple response
a) An alternative solution for accessing medical services during the pandemic
b) A solution that should be made permanent to increase accessibility to medical services
c) An alternative solution for certain categories of patients
d) An alternative solution for certain types of consultations
*e) another solution*____
f) I don't know / I don't answer

12) Propose alternatives to telemedicine to increase patient access to medical services:*
Arguments

***NB** *not measured - part of the qualitative section*

SECTION 3
Objective: Identifying possible solutions to prevent/combat the next wave or a future pandemic
Hypothesis: The more solutions we find, the greater the chance of preventing/combating the next wave or a future pandemic.

1) ***To prevent a new wave of SARS-CoV-2 infections or a possible future pandemic, do you consider it important to know the level of immunization of the population?***
Only one answer

a) *Yes, to a large extent*
b) *Yes, to some extent*
c) *Yes, to a small extent*
d) *No, not at all*
e) *I don't know/I don't answer.*

**** 2) If so, what measures to increase immunization are you considering?***
Arguments
____

3) ***To what extent do you consider information/education campaigns for the population to be useful/important to prevent/combat future infections?***
Only one answer
a) *To a very large extent*
b) *Largely*
c) *To a small extent*
d) *Not at all*
e) *I don't know/I don't answer.*

4) ***Do you agree to periodically perform RT-PCR testing of medical personnel?***
Only one answer
a) *Totally disagree*
b) *Partial disagreement*
c) *Partially agree*
d) *Totally agree*
e) *I don't know / I don't answer*

5) ***Do you consider testing for immunity acquired through disease or vaccination effective before a new vaccination?***
Only one answer
a) *Yes*
b) *Not at all*

**** 6) If you consider testing for immunity acquired through disease or vaccination before a new vaccination effective, please specify what its usefulness is?***
Arguments

____

7) ***How do you think medical/hospital units should be equipped during the pandemic?***
Multiple response
a) *Medical protective equipment*
b) *Medical equipment*

c) *Medicines*
d) *Medical devices*
e) *Creating specific compartments for infected patients*
f) *Qualified and trained personnel*
g) *Improved material and human resources of the units*
h) *Others:* ____

8) To what extent do you consider it necessary to regulate the legal framework regarding telemedicine / remote consultations?
Only one answer
a) To a very large extent
b) To a large extent
c) to a small extent
d) Not at all
f) I don't know/I don't answer

**** 9) Briefly present some opinions/perceptions or even recommendations from your professional experience during the COVID-19 pandemic***
Arguments

NB *not measured - part of the qualitative section

10) Socio-demographic data (Gender/residential area/age/specialty/professional degree/expertise in the field)

....
....
....
....
....

By completing this questionnaire, you agree that the data collected will be used in statistical analyses.

Date/month/year
This content is neither created nor endorsed by Google
Google forms

N.B. The survey was available online (docs.google.com) from the 27 January 2022 to the 28 February 2022.
